# Cholecystitis and Cardiomyopathy in an Immunocompetent Patient With Cytomegalovirus Infection: A Case Report

**DOI:** 10.7759/cureus.65418

**Published:** 2024-07-26

**Authors:** Hieu M Vo, Jerry M Sheppard

**Affiliations:** 1 Internal Medicine, William Carey University College of Osteopathic Medicine, Hattiesburg, USA; 2 Internal Medicine, Mississippi Baptist Medical Center, Jackson, USA

**Keywords:** viral cholecystitis, viral cardiomyopathy management, subclinical myocardial dysfunction, west nile virus infection, viral-induced myocarditis, epstein barr virus (ebv), cytomegalovirus-cmv

## Abstract

In this case report, we present a 53-year-old immunocompetent male exhibiting cholecystitis and cardiomyopathy related to cytomegalovirus (CMV) infection. The initial presentation pointed toward cholecystitis, including epigastric pain, chronic dysgeusia, dyspepsia, and cholelithiasis on ultrasound. A cholecystectomy was performed, and tissue analysis showed subacute cholecystitis. Postsurgical daily fever spikes prompted subsequent evaluation, which revealed CMV infection along with cardiomyopathy as evidenced by a reduced left ventricular ejection fraction, despite no suggestive clinical symptoms. Gastrointestinal symptoms, along with elevated liver enzymes, indicated possible congestive hepatopathy. Preceding symptoms also suggested a viral etiology, including a protracted fever and a possible transient Bell’s palsy. Medical management for viral myocarditis was initiated, and the patient has been followed closely after discharge. The case emphasizes the importance of considering viral etiology with comprehensive cardiac workup, even in the absence of overt cardiac symptoms but with abnormal liver enzymes. Surprisingly, the infectious workup showed positive West Nile virus (WNV) and Epstein-Barr virus (EBV) serology, indicating possible co-infection or cross-reactivity.

## Introduction

The prevalence of cytomegalovirus (CMV) infection ranges from 60% to 70% in developed countries to nearly 100% in developing regions, correlating positively with age [1]. In a U.S.-based study, CMV seroprevalence increased from 36% among six- to 11-year-olds to 91% in those over 80 [2]. Transmission occurs through body fluids, including sexual exposure, close contact, or perinatal exposure [3]. Primary infection in immunocompetent individuals often remains asymptomatic but may result in a self-limiting presentation. It can be life-threatening in immunocompromised patients. Common symptoms of primary CMV infection include prolonged fever, fatigue, night sweats, myalgia, and headache. Common laboratory findings include atypical lymphocytes, leukocytosis, and elevated serum transaminases [4]. CMV infection, although rare in immunocompetent individuals, can affect virtually every organ in the body, including gallbladder, heart, lung, brain, liver, and others. Similar presentations can occur due to Epstein-Bar virus (EBV) or West Nile virus (WNV), precluding definitive diagnosis due to possible co-infection or cross-reactivity with the testing reagents. 

This case report describes a patient with CMV infection manifesting as gallbladder and cardiac involvement. The viral workup also indicates likely past infections of EBV, but it is uncertain whether WNV positivity is due to an active or past infection. 

## Case presentation

**Figure 1:**
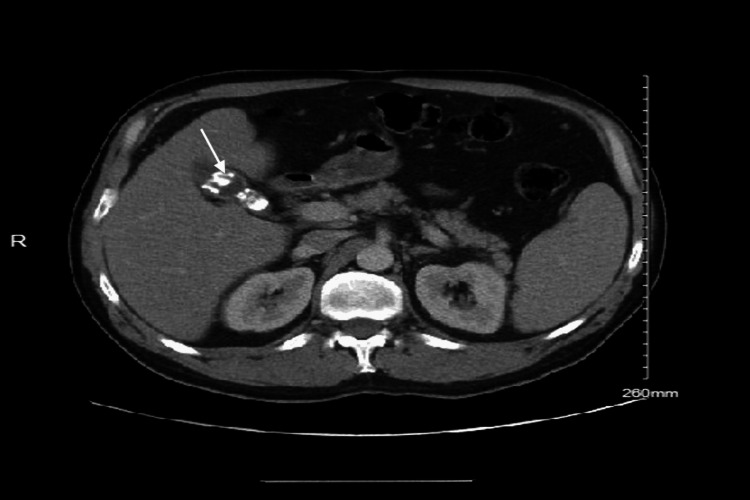
CT scan of the abdomen. The CT scan of the abdomen shows gallstones (white arrow) with the presence of fatty liver. There is no evidence of RUQ inflammation or bile duct dilation. CT: computed tomography, RUQ: right upper quadrant.

A 53-year-old male patient presented to the emergency department with a five-day history of epigastric abdominal pain, fever, nausea, and vomiting. He was in acute distress but alert. His vitals were: blood pressure 115/81, heart rate 95, respiratory rate 18, temperature 98.5°F (36.9°C), SpO_2_ 100%. Examination revealed right upper quadrant (RUQ) tenderness to palpation. Computed tomography (CT) of the abdomen with contrast showed fatty liver and cholelithiasis, but no sign of acute cholecystitis (Figure 1). There was no history of alcohol use disorder. Significant lab findings included elevated white blood cell count (WBC) with lymphocytic predominance and elevated liver enzymes with normal bilirubin (Table 1). He denied recent travel, tick bites or exposure to rabbits, cows, or other exotic animals. 

**Table 1:** Significant lab findings during the second emergency department visit. WBC: white blood cell count, AST: aspartate aminotransferase, ALT: alanine aminotransferase.

Test	Value	Reference range
WBC	14.4 K/uL	5-10 K/uL
AST	128 U/L	17-59 U/L
ALT	194 U/L	21-72 U/L
Alkaline phosphatase	305 U/L	38-126 U/L

Five days prior, he was evaluated in the local emergency department for a three-week history of fatigue, anorexia, cough, chills, diaphoresis, and fluctuating fever, peaking at 104°F. His vitals were: blood pressure 112/99, heart rate 119, respiratory rate 19, temperature 101.5°F (38.6°C), and SpO_2_ 98%. He was not in acute distress. Three weeks before the onset of symptoms, he experienced transient right-sided facial numbness and jaw movement difficulty. The exam at the time was significant for mild posterior oropharyngeal erythema. The screening for COVID-19, influenza, streptococcus infection, and mononucleosis was negative. Lab findings were significant for elevation in aspartate aminotransferase (AST), alanine aminotransferase (ALT), and alkaline phosphatase (ALP), but normal white blood cell count (WBC) (Table 2). Because he is a hunter, he was given doxycycline and Rocephin for a possible tick-borne illness. 

**Table 2:** Significant lab findings from the first emergency department visit, five days prior to the current presentation. WBC: white blood cell count, AST: aspartate aminotransferase, ALT: alanine aminotransferase.

Test	Value	Reference range
WBC	9.6 K/uL	5-10 K/uL
AST	206 U/L	17-59 U/L
ALT	285 U/L	21-72 U/L
Alkaline phosphatase	251 U/L	38-126 U/L

**Figure 2:**
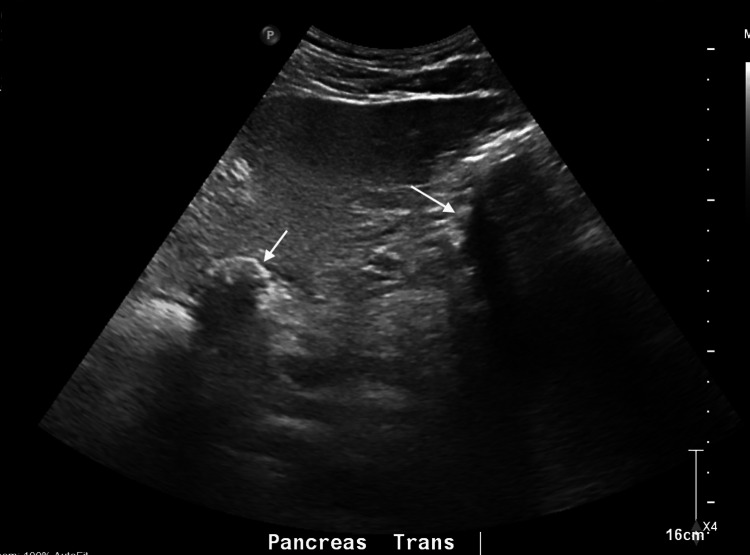
Ultrasound of the RUQ of the abdomen. There are stones (white arrows) with acoustic shadowing within the gallbladder and a fatty liver. RUQ: right upper quadrant.

**Figure 3:**
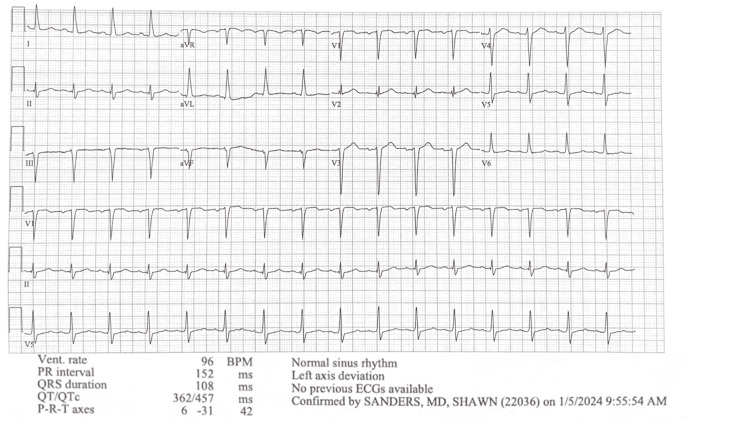
Electrocardiogram showed normal sinus rhythm with left axis deviation. Other findings were within normal limits.

Given his gastrointestinal symptoms, including dysgeusia, a long history of indigestion, and cholelithiasis on abdominal ultrasound (Figure 2), the patient underwent cholecystectomy, which revealed subacute and chronic cholecystitis from a pathology exam. Post-surgery, his abdominal pain and dysgeusia improved, but he continued to experience high-grade fever spikes, fatigue, diaphoresis, chills, anorexia, and persistent leukocytosis with lymphocytic predominance, along with conjunctival erythema. A subsequent extensive viral workup with cerebrospinal fluid (CSF) analysis showed a CMV IgM of 137 AU/mL (reference range: <35 AU/mL) and a CMV DNA viral load of 99.7 IU/mL (reference range: 0 IU/mL). EBV IgM was positive with an indeterminate EBV DNA viral load. Surprisingly, WNV IgM was also positive. The hepatitis panel was negative. An echocardiogram showed global hypokinesis with a left ventricular ejection fraction (LVEF) of 30-35%, despite the absence of dyspnea, orthopnea, syncope, or chest pain throughout the course of his illness up to this point. His wife reported that he preferred to sleep on the couch during this prolonged illness, but he is unsure if it is due to orthopnea. The electrocardiogram (Figure 3) and cardiac positron emission tomography (PET) were negative. Viral myocarditis was suspected to be the underlying cause of his abnormal echocardiogram. It is possible that his cholecystitis is also virally mediated. 

For viral myocarditis in immunocompetent patients, the mainstay of treatment is to manage acute heart failure with reduced ejection fraction, as antiviral therapy has uncertain efficacy [5]. Per guideline-directed medical therapy, he was put on Aldactone and Toprol, as these, along with angiotensin-converting enzyme inhibitors (ACEIs), have been shown to reduce the extent of myonecrosis, morbidity, and mortality [6,7]. The patient was discharged and will follow-up with his cardiologist, infectious disease specialist, and primary care physician. 

Post-discharge, the patient continued to have diaphoresis, low-grade fever controlled with Tylenol, and new-onset orthopnea. Repeated lab tests two weeks after discharge showed normalization of leukocytosis and liver function tests. A repeat echocardiogram showed no change in LVEF. He still had some fatigue. 

Three months after discharge, his echocardiogram still showed a reduced LVEF of 30-35%. He still reports increasing fatigue and a 7-lb weight loss in the past month. He denies lower extremity edema, orthopnea, paroxysmal nocturnal dyspnea, or palpitations. Long-term close follow-up is needed with medication titration since the recovery of cardiac function can take months or years. 

## Discussion

CMV infection is usually subclinical in the immunocompetent population, but it may manifest with flu-like symptoms and can involve multiple organs. It should be part of the differential diagnosis in case of protracted, fluctuating fever and other flu-like symptoms. Other findings consistent with a viral etiology include a recent history of possible mild right-sided Bell's palsy, isolated liver enzyme elevation, and lymphocyte-predominant leukocytosis. His history of spending time and sharing drinks with his grandchildren can be a potential exposure factor. While the initial presentation at the ED suggested possible cholecystitis from cholelithiasis, the preceding protracted fever and flu-like symptoms indicated a viral infection as a likely etiology. The echocardiogram’s findings of moderate-severe cardiomyopathy with an LVEF of 30-35% and global hypokinesis were unexpected, given the absence of cardiac symptoms, including chest pain, dyspnea, orthopnea, or syncope, until post-discharge. The echocardiogram was ordered due to isolated liver enzyme elevation and fatigue, which could be due to congestive hepatopathy and low cardiac output. This underscores the potential need to include an echocardiogram in a viral infection workup, even in the absence of obvious cardiac symptoms, which could be delayed. Retrospectively, the cholecystitis change seen in the pathology report could be attributed to chronic congestion due to a reduced ejection fraction, although we do not know how long the patient has had cardiomyopathy. 

The case demonstrates the diagnostic dilemma in determining the true cause of cardiomyopathy and cholecystitis without a pathology study, due to the multiple positive results in the viral panel. The high CMV viral load indicates active infection, while the positive EBV IgM with indeterminate EBV polymerase chain reaction (PCR) suggests possible cross-reactivity or past infection. WNV IgM can persist for up to eight years in 23% of study participants [8]. Together with a case report [9] demonstrating the presence of viral cardiomyopathy with an isolated positive WNV IgM, WNV could be the cause of this patient’s presentation. In addition, protracted flu-like symptoms are common in both CMV and WNV infections. Thus, an invasive biopsy may be necessary for a definitive diagnosis, but it is not often performed.

## Conclusions

The expected outcome of acute myocarditis depends on the severity of the initial presentation. With mild disease, patients usually have a good prognosis with partial or complete recovery. The fulminant disease often leads to cardiogenic shock with high mortality. Some may continue to have subclinical disease, which later becomes more symptomatic with dilated cardiomyopathy. In viral myocarditis, early treatment with ACEIs and BBs can reduce morbidity and mortality. Close follow-up with a cardiologist is crucial since recovery can take months to years.

This case illustrates the potential complexity in diagnosing and managing patient with CMV infection due to its cardiac and gallbladder involvement. The multiple positive lab findings for CMV, EBV, and WNV may represent possible co-infection or cross-reactivity with diagnostic reagents. Fortunately, the lack of a definitive diagnosis does not change the general management needed to optimize recovery in viral cardiomyopathy. 
